# Some More Incompatible Prescriptions

**Published:** 1909-07-03

**Authors:** 


### THERAPEUTICS AND PHARMACY.

### SOME MORE INCOMPATIBLE PRESCRIPTIONS.

Actual prescriptions that prove to be unsightly, dangerous, or otherwise unsuitable when dispensed are always of interest to the physician, especially if the causes of their imperfections can be avoided on subsequent occasions. The following are some further examples of incompatible prescriptions collected from the *Pharmaceutical Journal*:—

| | | | |
|---|---|---|---|
| ℞ | Potassii Chloratis ... ... ... | | gr. xx. |
| | Calcii Chloridi ... ... ... ... | | ʒj. |
| | Acidi Sulphurosi ... ... ... | | ʒij. |
| | Aquam chloroformi ... ... | ad | ℥iij. |

Misce. Fiat Mistura.

When compounded as written a copious precipitate forms when the mixture has been standing about two hours or so. This is owing to an interaction between the potassium chlorate and the sulphurous acid, as the result of which sulphuric acid is formed. The sulphuric acid in its turn reacts with the calcium chloride, and relatively insoluble calcium sulphate is produced, together with free hydrochloric acid also.

| | | | |
|---|---|---|---|
| ℞ | Potassii Iodidi ... ... ... ... | | ʒij. |
| | Tincturæ Ferri Perchloridi ... ... | | ʒiij. |
| | Infusum Quassiæ ... ... | ad | ℥vj. |

Misce. Fiat Mistura.
Sig.: A tablespoonful three times a day.

In this case there is the old difficulty of liberation of iodine by interaction between the potassium iodide and the perchloride of iron. Iron and ammonium citrate might be used instead of the latter.

| | | | |
|---|---|---|---|
| ℞ | Plumbi Acetatis ... ... ... | | gr. xxx. |
| | Acidi Acetici diluti ... ... ... | | ʒij. |
| | Syrupi Tolutani ... ... ... | | ʒiv. |
| | Syrupi Limonis ... ... ... ... | | ℥iss. |
| | Aquam Cinnamomi ... ... | ad | ℥vj. |

Misce. Fiat Mistura.

This mixture gives a white precipitate soon after it is made up, owing to the formation of lead citrate by interaction between the lead acetate and the citric acid in the lemon syrup. The latter is only required for flavouring purposes, and the same end could be attained by prescribing syrupus simplex and tinctura limonis without any lead citrate coming down at all.

| | | | |
|---|---|---|---|
| ℞ | Potassii Iodidi ... ... ... ... | | ʒij. |
| | Liquoris Strychninæ ... ... ... | | ʒiss. |
| | Aquam ... ... ... ... | ad | ℥iij. |

Misce. Fiat Mistura.
Sig.: A dessertspoonful three times a day.

This is a dangerous prescription, owing to the risk there is that the comparatively insoluble strychnine hydriodide will become thrown out in the form of crystals, and settle at the bottom.

| | | | |
|---|---|---|---|
| ℞ | Atropinæ Sulphatis ... ... ... | | gr. j. |
| | *Strychninæ Hydrochloridi ... ... | | gr. ij. |
| | Acidi Salicylici ... ... ... ... | | gr. iij. |
| | Sodii Biboratis ... ... ... ... | | gr. ij. |
| | Aquæ destillatæ ... ... ... | | ℥iss. |

Misce. Fiat Mistura.

In this case prompt abundant precipitation of the alkaloids atropine and strychnine is brought about by the sodium biborate, and the dangers that might ensue are obvious.

| | | | |
|---|---|---|---|
| ℞ | Ammonii Bromidi ... ... ... | | ʒiiss. |
| | Magnesii Sulphatis ... ... ... | | ℥vj. |
| | Tincturæ Nucis Vomicæ ... ... | | ʒj. |
| | Spiritûs-Chloroformi ... ... ... | | ʒij. |
| | Aquam ... ... ... ... | ad | ℥iij. |

Misce. Fiat Mistura.

Immediately this mixture is made up a dense crystalline precipitate is thrown down. This consists of some magnesium salt. The difficulty can be obviated at once by adding water to 6 oz. instead of only to 3 oz., in which case the patient will be told to take double the dose.

